# Study of initiatives to address shortage of specialists for emergency obstetric care in Maharashtra, India

**DOI:** 10.1186/1753-6561-6-S1-O1

**Published:** 2012-01-16

**Authors:** Sarika Chaturvedi, Bharat Randive

**Affiliations:** 1 Foundation for Research in Community Health, Pune, India

## Introduction

Emergency obstetric care (EmOC) is one of the concrete “service guarantees” of the National Rural Health Mission (NRHM) launched by the Government of India in 2005. However the Indian public health system suffers from a severe shortage of specialists to deliver EmOC.

As a part of a larger study of EmOC provision in Indian state of Maharashtra, we studied the government's strategies to expand the network of skilled providers through task shifting for anaesthesia and obstetric services and models of public private partnerships. In this study, we address the gap in our understanding of the potential of such strategies in providing EmOC, their influence on the size and distribution of obstetric and anaesthesia providers and the related service uptake.

## Methods

We used a mix of quantitative and qualitative methods. We conducted a facility survey of all secondary and tertiary health care facilities (44) in 3 districts. We interviewed medical superintendents and specialist obstetric service providers at public facilities (20) in these districts, selected private obstetricians and anaesthetists (15) and the district health officials and programme managers. We mapped the location of the private obstetricians in the three districts.

In order to study access, we selected 6 blocks in the districts where we randomly selected 60 health sub-centres and conducted a community level survey in the 272 villages covered by these centres. In these villages we listed all women (1833) who were either below poverty line (BPL) or belonged to scheduled caste (SC) or scheduled tribe (ST) and who delivered their first or second live baby within one year from the survey. We identified women who experienced obstetric complication/s and we interviewed 120 such women selected through maximum variation sampling.

## Results

Of the 44 public facilities we studied, we found that 20 (45%) have a qualified obstetrician/s, 13 (30%) have a qualified anaesthetist/s while 77% do not have either/ both of these specialists. The utilisation of the specialist skills of the serving obstetricians is low – the 25 obstetricians working at sub-district level performed 34 caesarean sections during six months.

The number of obstetricians working in the private sector in these districts translates to an availability of one obstetrician for 23,000 population in Amravati, one for 21,000 in Satara and one for 48,000 in Nandurbar district. The corresponding availability of obstetricians in the public sector was one for 325,000 in Amaravati, one for 187,000 in Satara, and 1 for 163,000 population in Nandurbar district. Half of the 260 private obstetricians in the study district work in rural areas. Our findings contradict the assumption that there is an overall shortage of obstetricians. In the interviews with private specialists, they raised concerns in rendering services in government facilities.

For 50% of the public facilities with potential for contracting in specialists, the nearest private specialist is located more than 30 km (range 30 km to 100 km). Contracting in has been undertaken at seven facilities and these are mostly places where the private specialist is located in the same town. Though contracting in has provided specialists for fixed duration it has not influenced the provision of obstetric services in emergencies.

The interviews with the private specialists revealed their expectations and the interviews with administrators and managers brought forward the operational issues in executing the contracting in strategy.

Regarding task shifting, we met the 2 graduate medical officers trained in comprehensive EmOC services and the 3 others trained in providing anaesthesia (for caesarean section). None of these have independently conducted a caesarean section or have administered anaesthesia for it. Interviews with these providers revealed the reasons for non-performance.

Unavailability of caesarean section in public facilities has effect on general population. Our community level survey shows that 8% of the women had undergone a caesarean section and 57% of these surgeries were in the private sector. The cost incurred for caesarean section in the private sector ranges from INR 10,000 (USD 214.7) to INR 30,000 (USD 644.2). In the absence of caesarean services at 80% of the community health centres (CHC), the next public facility with these services is located more than 60 km for over half of the these CHCs.

## Discussion

We discuss the appropriateness of initiatives for addressing specialist shortage in states like Maharashtra that have a relatively higher concentration of specialists and the limitations of contracting in and task shifting strategies. We highlight the neglect towards providing the required working environment for these cadres. Aiming for a sustainable solution, we recommend the areas for essential changes in human resource policies to attract and retain specialists in the system.

